# A case report of prostate adenocarcinoma with leptomeningeal carcinomatosis and intracerebral metastasis

**DOI:** 10.1002/ccr3.5000

**Published:** 2021-10-28

**Authors:** Danial Fazilat‐Panah, Nahid Ahmadi, Dariush Moslemi, Masoumeh Karimi, Alireza Taji, Mansoureh Dehghani, Ali Emadi Torghabeh, Zahra Keshtpour Amlashi, Mohammadreza Saghafi, Maedeh Alsadat Fatemi

**Affiliations:** ^1^ Cancer Research Center Babol University of Medical Sciences Babol Iran; ^2^ Neyshabur University of Medical Sciences Neyshabur Iran; ^3^ Cancer Research Center Mashhad University of Medical Sciences Mashhad Iran; ^4^ Department of Radiotherapy and Oncology Faculty of Medicine Mashhad University of Medical Sciences Mashhad Iran; ^5^ Hamadan University of Medical Sciences Hamadan Iran; ^6^ Qazvin University of Medical Sciences Qazvin Iran

**Keywords:** CNS metastasis, leptomeningeal carcinomatosis, prostate cancer

## Abstract

Despite the fact that prostate cancer is the most prevalent cancer in men, metastases to the central nervous system including leptomeningeal involvement by prostate carcinoma is a rare event. The prognosis of metastatic prostate cancer is very poor due to lack of CNS penetrating therapeutic agents.

## Graphical abstract

Despite the fact that prostate cancer is the most prevalent cancer in men, metastases to the central nervous system including leptomeningeal involvement by prostate carcinoma are a rare event. The prognosis of metastatic prostate cancer is very poor due to lack of CNS penetrating therapeutic agents.

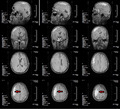

## 1 INTRODUCTION

Despite the prostate cancer is the most prevalent men cancer, metastases to the central nervous system including leptomeningeal involvement by prostate carcinoma are a rare event. Here, we detected leptomeningeal carcinomatosis based on MRI findings in a 67‐year‐old patient with castration‐resistant metastatic prostate cancer who presented paraplegia and paresthesia of both limbs.

Prostate cancer is second only to lung cancer as a leading cause of cancer‐related death in men.^1^ Most men with prostate cancer have asymptomatic and indolent disease. In advanced stages, the most common locations of metastasis from the prostate are bone, lung, and liver.^2^ Central nervous system (CNS) complications commonly occur in the late stages of advanced disease. In rare occasions, leptomeningeal carcinomatosis (LC) can occur in patients with prostate cancer.^3^, ^4^ In addition to the metastatic disease, paraneoplastic syndromes such as neuropathies, cerebellar ataxia, and limbic and brainstem encephalitis, may also occur.^5^, ^6^ Here, we report a patient with metastatic prostate cancer who developed CNS symptoms, and the MRI study of the brain revealed leptomeningeal metastasis.

## 2 CASE

**FIGURE 1:**
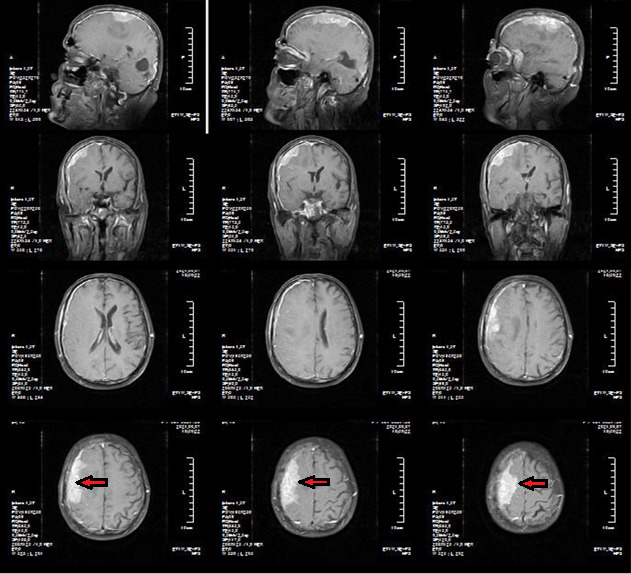
Brain MRI obtained in sagittal, axial, and coronal planes. On post‐contrast MRI images, there are diffuse enhancement of leptomeninges. There is also an enhancing mass at the upper aspect of the parietal lobe (red arrow)

The case is a 67‐year‐old male patient who was first admitted to the urology department with a history of dysuria and dribbling at the termination of urination, 3 years ago. There were no other significant medical or familial histories. Further assessments showed an elevated prostate‐specific antigen (PSA, 50 ng/mL) in addition to normal values for complete blood count, kidney, and liver function, alkaline phosphatase, and lactate dehydrogenase. A computed tomography scan (CT) of the chest, abdomen, and pelvis with and without intravenous contrast was obtained showing an enlarged prostate without any regional lymph‐nodes involvement or distant metastasis. A 12‐core transrectal ultrasound‐guided prostate needle biopsy (TRUS) revealed a high‐grade adenocarcinoma of the prostate (the Gleason score 4 + 4 = 8). The patient underwent a whole body bone scan demonstrating no clear evidence of bone metastasis. As a patient with high‐risk prostate cancer, the patient received 2 months of neoadjuvant gonadotropin‐releasing hormone (GnRH) agonist. Subsequently, definitive radiotherapy of whole pelvis (50 gray [Gy]/25 fractions [frs]) with a tumor bed boost of 20 Gy in 10 frs was prescribed, along with a concurrent GnRH agonist. After completion of radiotherapy, GnRH agonist was continued and a PSA nadir of 1 ng/mL was recorded. Five months later, the patient underwent a whole body bone scan due to generalized bone pain showing wide‐spread bone metastases of thoracolumbar vertebral spine. The serum level of the PSA raised to 280 ng/mL, while the serum level of the testosterone was 5 ng/dL proving a diagnosis of castration‐resistant prostate cancer. At this point, zoledronic acid was prescribed 4 mg monthly and palliative radiotherapy to the symptomatic regions was performed (30 Gy/10 frs). After the completion of palliative radiotherapy, the patient was treated with docetaxel (75 mg/m^2^) every 3 weeks and daily prednisolone (5 mg twice a day) for ten courses resulting in significant reduction of serum level of PSA to 5 ng/mL. After 5 months of follow‐up, the serum level of PSA raised to 260 ng/mL and the patient developed paraplegia and paresthesia of both limbs. A brain magnetic resonance imaging (MRI) with and without contrast showed diffuse high signal intensity within the subarachnoid space of the sulci and cisterns on post‐contrast T1‐weighted images (Figure 1). The patient refused lumbar puncture (LP) for cerebrospinal fluid (CSF) examination.

Patient received whole brain radiotherapy (WBRT) at a dose of 30 Gy in 10 frs. After WBRT, abiraterone acetate (1,000 mg daily) plus prednisolone (5 mg twice daily) was prescribed; however, no significant response was detected. Therefore, a rechallenge by docetaxel plus prednisolone was considered. After five courses, the patient had a stable disease and the level of PSA was decreased to 10 ng/mL.

## 3 DISCUSSION

Leptomeningeal carcinomatosis (LC) is an uncommon complication of cancer in which the disease metastasizes to the meninges; the term “leptomeninges” refers to the combination of the two meninges, the arachnoid and the pia mater, between which circulates the cerebrospinal fluid. It is estimated that LC occurs in 5% of cancer patients and is most often terminal. Leptomeningeal spread occurs more with hematologic tumors, such as leukemia, at an incidence of 10–15%, and much less frequently with solid tumors at an incidence of 1–5%, the most common being breast, lung, melanoma, gastrointestinal, and primary CNS tumors.^7^, ^8^ LC is a rare complication in the course of prostate cancer. An example of the prevalence of LC can be provided by a 33‐year retrospective review published by the University of Texas MD Anderson Cancer Center. The study aimed to detect all patients with LC originating from primary GU cancer. Of the 93,960 GU cancer patients, a mere 31 cases of LC were identified, which is approximately 0.03%. Of those that were identified, only 7 were prostate in origin.^9^ The most common intracranial sights of metastatic prostate cancer are the leptomeninges (67%), cerebrum (25%), and cerebellum (8%).^10^ Typical presenting symptoms of LC include altered mental status, headache, nausea, vomiting, and cranial nerve palsies.^11^, ^12^ The patient in this report presented with paraplegia and paresthesia of both limbs. LC diagnosis is challenging given the poor sensitivity of diagnostic modalities. Following detailed physical examination, the patient should undergo a contrast MRI. However, the reported sensitivity is highly variable across the literature ranging from 77 to 100%. As in our patient, diffuse enhancement of the leptomeninges was discovered. Logically, the next step would be an LP. The most definitive tool in the diagnosis of LC is the LP, searching for cytological identification of malignant cells in the CSF.^4^ Given that CSF cytology can have false‐negative rates as high as 35%, currently, MRI with contrast is considered the most sensitive imaging modality, with almost 100% sensitivity. Gadolinium‐enhanced T1‐weighted MRI sequences are considered the best noninvasive means of detecting LC. Diagnostic findings include leptomeningeal enhancement of the brain, spinal cord, cauda equina, or sub‐ependymal areas. The enhancement may extend into the sulci of the cerebrum or folia of the cerebellum.^13^ Experts suggest that when there is strong evidence of LC on MRI, cytological confirmation is not necessary, and physicians can proceed with the treatment.^14^ Our patient refused LP and treatment began based on clinical findings and MRI. There is currently no standard treatment for prostate cancer LC and a decision is best made by a multidisciplinary team. Quality of life should be a focal point and the ultimate treatment goal without exception. Treatment options suggested by the literature include hormonal treatment (in castration‐sensitive prostate cancer), corticosteroids, debulking surgery, WBRT, and intrathecal chemotherapy. However, all of these treatments have been associated with poor outcomes.^15^ The present patient was treated with WBRT followed by ineffective hormonal treatment, so he was rechallenged with systemic chemotherapy resulting in favorable clinical and biological responses. Longer follow‐up and further studies are preferable to come to a conclusion about an standard approach, especially as the prevalence of LC cases tends to increase as a result of more effective treatments and extended survival in prostate cancer patients.

Currently available therapeutic agents in the management of metastatic and locally advanced prostate cancer do not cross blood‐brain barrier adequately. It seems that improvement of radiotherapy techniques beside finding more about the tumor biology and inventing new targeted drugs might help local and systemic control of patients with prostate adenocarcinoma with leptomeningeal carcinomatosis and intracerebral metastasis.^16^, ^17^, ^18^, ^19^, ^20^, ^21^, ^22^

## 4 CONCLUSION

In prostate cancer patients who present with focal neurological symptoms any time during the course of metastatic phase, leptomeningeal carcinomatosis should be taken into consideration, although it is rare. Timely diagnosis and proper treatment approach may prevent symptom exacerbation and improve quality of life.

## CONFLICTS OF INTEREST

Authors declare that they have no conflicts of interest.

## AUTHOR CONTRIBUTIONS

All authors contributed equally.

## ETHICAL APPROVAL

The study was approved by Babol University of medical Sciences.

## CONSENT

An informed written consent form was obtained from patient.

## Data Availability

The data sets used and/or analyzed during the current study are available from the corresponding authors per request.
